# Acute Coronary Syndrome Management in Older Patients: A Dual-Center Retrospective Cohort Study

**DOI:** 10.3390/medicina61081436

**Published:** 2025-08-09

**Authors:** Karlo Gjuras, Ivana Jurin, Hrvoje Jurin, Eduard Margetić, Boško Skorić, Joško Bulum, Irzal Hadžibegović, Ivan Zeljković, Marin Pavlov, Šime Manola, Kristina Marić Bešić

**Affiliations:** 1 Department of Family Medicine, Health Centre Bjelovar-Bilogora County, 43000 Bjelovar, Croatia; karlogjuras4@gmail.com; 2 Department of Cardiovascular Medicine, Dubrava University Hospital, 10000 Zagreb, Croatia; ivanajurin1912@gmail.com (I.J.); irzalh@gmail.com (I.H.); ivanzeljkov@gmail.com (I.Z.); marin.pavlov@gmail.com (M.P.); 3 Department of Cardiovascular Diseases, University Hospital Centre Zagreb, 10000 Zagreb, Croatia; hrvoje.jurin@gmail.com (H.J.); eduard.margetic@gmail.com (E.M.); bskoric3@yahoo.com (B.S.); jbulum@gmail.com (J.B.); kmaricbesic@gmail.com (K.M.B.); 4 School of Medicine, University of Zagreb, 10000 Zagreb, Croatia; 5 Professional Undergraduate Study Physiotherapy, University North, 48000 Koprivnica, Croatia

**Keywords:** acute coronary syndrome, older patients, management, invasive treatment

## Abstract

*Background and Objectives*: Older patients with ACS are less frequently treated with an invasive strategy, which may negatively impact their survival. This study aimed to investigate treatment approaches in elderly ACS patients and compare outcomes between invasively and conservatively treated groups. *Materials and Methods*: This retrospective study included consecutive patients aged 80 or older who presented with ACS at two tertiary institutions from November 2018 to October 2023. The invasive group consisted of patients who underwent percutaneous or surgical revascularization. The conservative strategy was defined as guideline-directed medical therapy only. The primary outcome was all-cause mortality during the six-month follow-up. Secondary outcomes were recurrent MI and CVI. *Results*: Among 670 ACS patients with a median age of 83 years (81–86) and 50.6% women, 429 (64%) were treated with an invasive strategy, and 241 (36%) were treated with a conservative strategy. A total of 176 (26%) patients died during the six-month follow-up period, with significantly higher mortality observed in the conservatively treated group compared to the invasively treated group (ACS: 37.8% vs. 19.3%, *p* < 0.001; STEMI: 49.4% vs. 26.8%, *p* < 0.001; NSTE-ACS: 32.1% vs. 10.9%, *p* < 0.001). Recurrent MI was documented in 2.5% of patients, while CVI occurred in 1.2%, with no difference between the treatment groups. Multivariable regression analysis identified invasive strategy (HR = 0.48; 95% CI: 0.33–0.71; *p* < 0.001) as a positive predictor of six-month survival in ACS patients. *Conclusions*: The invasive treatment strategy was associated with lower mortality in older ACS patients, regardless of the type of ACS. The incidence of recurrent MI and CVI did not differ between groups treated with different therapeutic approaches.

## 1. Introduction

With the progressive aging of the global population, the proportion of elderly individuals presenting with acute coronary syndrome (ACS) is steadily increasing. It is estimated that nearly one-third of all patients hospitalized with ACS are aged 75 years or older, and mortality in this age group is twice as high compared to younger patients [1,2]. Despite their elevated risk profile, data from clinical registries consistently demonstrate that elderly patients are less likely to receive an invasive treatment strategy. This underutilization of invasive interventions in older adults has been associated with poorer survival outcomes [3].

Aging is accompanied by numerous pathophysiological changes that adversely affect cardiovascular health. These include increased arterial stiffness, myocardial hypertrophy, endothelial dysfunction resulting in impaired vasodilation, diminished responsiveness to β-adrenergic stimulation, and enhanced atherogenesis. Collectively, these changes contribute to an imbalance between myocardial oxygen supply and demand, predisposing elderly individuals to type 2 myocardial infarction [1,4,5]. Moreover, advanced age is associated with dysregulation of the thrombogenic and fibrinolytic systems, increasing the risk of both ischemic and hemorrhagic events in this vulnerable population [6]. In addition to these physiological alterations, there is a high prevalence of geriatric syndromes among older adults. These syndromes are characterized by multimorbidity, frailty, cognitive, physical, and sensory impairments, increased propensity for falls, and polypharmacy [7,8]. Consequently, the clinical presentation of ACS in elderly patients is often atypical. Nearly half of older patients present without classic symptoms [9], while electrocardiogram interpretation is complicated by pre-existing abnormalities in approximately 70% of cases [10]. Furthermore, the specificity of high-sensitivity cardiac troponin assays is reduced in this population, further delaying the timely diagnosis and initiation of appropriate therapy [11,12].

Despite their high risk, elderly patients are frequently underrepresented or excluded from randomized controlled trials (RCTs) due to their age, multiple comorbidities, and limited life expectancy [13,14]. Even studies specifically designed to evaluate outcomes in older adults often apply stringent exclusion criteria, limiting the generalizability of their findings to the broader elderly population [15]. Current guidelines from the European Society of Cardiology (ESC) recommend that elderly patients with ACS should receive treatment strategies similar to those recommended for younger patients. However, in older adults with geriatric syndromes—who represent the majority of this population—a holistic and individualized treatment approach is advocated [16].

To date, six small prospective RCTs have investigated the outcomes of invasive versus conservative treatment strategies in elderly patients with non-ST-elevation acute coronary syndrome (NSTE-ACS) or non-ST-elevation myocardial infarction (NSTEMI) [15,17,18,19,20,21]. A meta-analysis of these trials demonstrated no significant reduction in all-cause or cardiovascular mortality with an invasive approach compared to conservative management. However, invasive treatment was associated with a significantly lower incidence of recurrent myocardial infarction (MI) and repeat revascularization procedures [22]. The largest and most recent of these trials, the SENIOR-RITA study, reported findings consistent with those of the preceding meta-analysis [23]. In contrast, data from retrospective observational studies consistently suggest a survival advantage for elderly ACS patients managed with an invasive strategy compared to those treated conservatively [24,25,26,27,28,29,30].

The aim of this study was to investigate the impact of invasive and conservative treatment strategies on mortality, as well as the incidence of recurrent MI and cerebrovascular insult (CVI) during a six-month follow-up period in elderly patients with ACS. In addition, we compared demographic and clinical characteristics between the different treatment groups and identified factors influencing the selection of an invasive strategy, as well as predictors of six-month survival in this high-risk population.

## 2. Materials and Methods

### 2.1. Patients and Data Collection

This retrospective study included 670 patients aged 80 years or older admitted with a diagnosis of ACS to either University Hospital Centre Zagreb (Zagreb, Croatia) or University Hospital Dubrava (Zagreb, Croatia) between November 2018 and October 2023. Data on demographic characteristics, medical history, laboratory results, and pharmacological treatment at admission and discharge were collected through the hospital information system, following regulations governing personal data protection.

**Figure 1:**
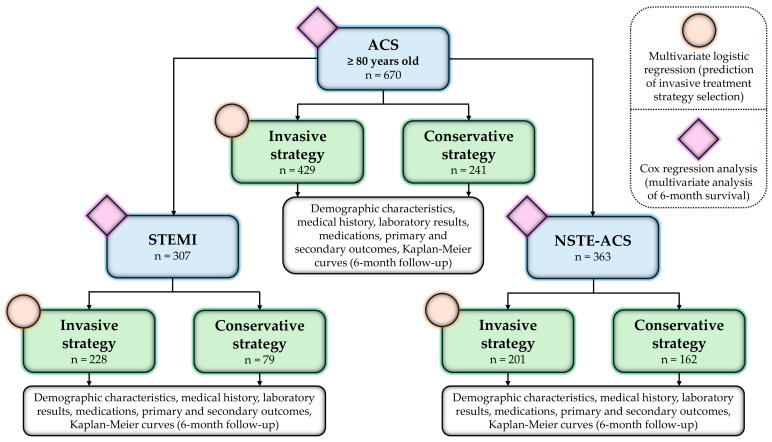
Study design and workflow of a retrospective study investigating the association between different treatment strategies in older patients with acute coronary syndrome and clinical outcomes.

All patients diagnosed with ACS were stratified into two groups based on treatment strategy: the invasive group and the conservative group. In addition, patients were classified according to the type of ACS into either the ST-elevation myocardial infarction (STEMI) group or the NSTE-ACS group. Each diagnostic category was further subdivided based on the treatment approach—into invasive and conservative subgroups (Figure 1). Diagnoses were made according to the ESC guidelines valid at the time of patient admission [16,31,32]. In cases where patients were hospitalized multiple times for ACS during the study period, only the first hospitalization was included in the analysis.

The study was approved by the Ethics Committee of the University Hospital Centre Zagreb.

### 2.2. Treatment Strategies and Outcomes

The invasive treatment strategy was defined as either primary percutaneous coronary intervention or coronary artery bypass grafting, combined with optimal medical therapy. The conservative treatment strategy was defined as guideline-directed medical therapy with or without coronary angiography.

The primary outcome was defined as all-cause mortality. Secondary outcomes included recurrent MI and CVI. A major adverse cardiovascular event (MACE) was defined as death, nonfatal stroke, or nonfatal myocardial infarction. Patients were followed for six months.

### 2.3. Statistical Analysis

The normality of distribution for continuous variables was assessed using the Shapiro–Wilk test. Variables with a normal distribution were expressed as means ± standard deviations (SD) and compared between groups using the parametric Student’s t-test. Variables with non-normal distribution were presented as medians with interquartile ranges (IQR) and compared using the non-parametric Mann–Whitney test. Categorical variables were presented as frequencies and percentages and compared between groups using the chi-square test.

We conducted 1:1 nearest-neighbor propensity score matching (PSM) without replacement using a caliper width of 0.1 standard deviations of the logit of the propensity score. Covariates included age, sex, diabetes, left ventricular ejection fraction (LVEF) ≤ 40%, admission hemoglobin ≤ 100 g/L, moderate or severe aortic stenosis, atrial fibrillation, prior MI, and previous use of angiotensin-converting enzyme (ACE) inhibitor or angiotensin receptor blocker (ARB). After matching, all standardized mean differences were below 0.10 except age (−0.156), indicating good covariate balance. A total of 436 patients (218 in each group) were included in the matched cohort. Propensity score estimation and matching were performed in R (version 4.5.1, R Core Team, 2024) using the MatchIt package.

Logistic regression analysis was performed to identify independent predictors of invasive treatment selection (odds ratios [OR] with 95% confidence intervals [CI]). The variables included in the analysis were demographic characteristics, clinical and laboratory findings during hospitalization, and therapy at admission. Variables with a *p*-value < 0.10 in univariate analysis were entered into multivariate logistic regression models. Kaplan–Meier survival curves were used to illustrate survival probabilities between groups, and differences were assessed using the log-rank test. Multivariate Cox proportional hazards regression analysis was conducted to identify independent predictors of survival (hazard ratios [HR] with 95% CI). The variables included in the model were age, treatment strategy, sex, aortic stenosis, atrial fibrillation, previous MI, diabetes mellitus, LVEF ≤ 40%, hemoglobin ≤ 100 g/L, and therapy at admission. Cox regression was performed on both unmatched and matched data.

A two-tailed *p*-value < 0.05 was considered statistically significant for all tests. All statistical analyses were performed using the Jamovi statistical software package (version 2.5.5, The Jamovi Project, 2024), except for the PSM analysis.

## 3. Results

### 3.1. Demographic, Clinical, and Laboratory Characteristics

#### 3.1.1. Acute Coronary Syndrome

The study included 670 patients diagnosed with ACS (Table 1). Of these, 429 (64%) underwent invasive management, while 241 (36%) were treated conservatively. The median age was 83 years (IQR 81–86), with older patients more frequently receiving conservative treatment (*p* < 0.001). Gender distribution was balanced overall, although women were less likely to be treated invasively compared to men (204/339 [60.2%] vs. 225/331 [68.0%], *p* = 0.035).

Hyperlipidemia was more common in the invasive group (49.9% vs. 40.2%, *p* = 0.016), consistent with higher total and low-density lipoprotein (LDL) cholesterol levels. There were no significant differences between groups in the prevalence of hypertension, diabetes mellitus, malignancy, prior myocardial infarction, or stroke. Atrial fibrillation (34.0% vs. 24.9%, *p* = 0.012) and aortic stenosis (18.3% vs. 7.7%, *p* < 0.001) were more frequently observed in the conservatively managed group, which also presented with higher resting heart rates at admission (78 vs. 80 bpm, *p* = 0.003) and more often had a LVEF below 40% during hospitalization (35.3% vs. 26.8%, *p* = 0.022). These patients were more frequently on chronic therapy with antiplatelets, beta-blockers, and statins. Laboratory results in the conservative group showed significantly lower hemoglobin levels, nearly double the N-terminal pro–B-type natriuretic peptide (NT-proBNP), and triple the C-reactive protein (CRP) concentrations. There were no significant differences in major bleeding events (*p* = 0.335).

Table 2 presents the characteristics of older patients with ACS, stratified by treatment strategy after propensity score matching.

STEMI was diagnosed in 307 (45.8%) of patients, while 363 (54.2%) had NSTE-ACS. Among NSTE-ACS patients, NSTEMI was diagnosed in 325 (89.5%) cases. Differences in demographic and clinical characteristics, treatment strategies, and primary and secondary outcomes according to ACS type are summarized in Table 3.

#### 3.1.2. ST-Elevation Myocardial Infarction

Among 307 STEMI patients, 228 (74.3%) received invasive and 79 (25.7%) conservative treatment (Table 4). Older patients were more often managed conservatively (*p* = 0.038), and there was no significant difference between sexes regarding treatment approach (*p* = 0.073).

Atrial fibrillation (26.3% vs. 43.0%, *p* = 0.005) and beta-blocker use at admission (34.5% vs. 50.6%, *p* = 0.013) were less common in the invasive group. These patients also had lower diastolic blood pressure on presentation. LVEF ≤ 40% was more frequently seen in the conservative group (48.1% vs. 33.3%, *p* = 0.019), which also experienced a higher incidence of cardiogenic shock during hospitalization (17.7% vs. 8.8%, *p* = 0.029). NT-proBNP and CRP levels were significantly higher in this group.

#### 3.1.3. Non-ST-Elevation Acute Coronary Syndrome

There were 363 patients with NSTE-ACS, with 201 (55.4%) receiving invasive and 162 (44.6%) conservative management (Table 5). As in the STEMI group, older patients were more frequently treated conservatively (*p* < 0.001). However, in this subgroup, a significant gender difference was observed—women were less likely to receive invasive treatment compared to men (71/151 [47.0%] vs. 130/212 [61.3%], *p* = 0.007).

Patients treated invasively more often had a history of hyperlipidemia and peripheral artery disease, whereas chronic obstructive pulmonary disease (COPD) and aortic stenosis were more frequent in the conservative group. At admission, conservatively treated patients had higher diastolic blood pressure, and during hospitalization, higher NT-proBNP and CRP levels, as well as more frequent LVEF ≤ 40%.

### 3.2. Treatment Strategies

#### 3.2.1. Predictors of Invasive Strategy

Multivariate logistic regression identified age and CRP as independent predictors of treatment strategy in ACS patients (Table 6). In the NSTE-ACS group, age was a strong independent predictor of conservative management, with moderate-to-severe aortic stenosis (OR = 0.45; 95% CI: 0.23–0.87; *p* = 0.018), COPD (OR = 0.29; 95% CI: 0.09–0.92; *p* = 0.035), and hemoglobin ≤ 100 g/L during hospitalization (OR = 0.44; 95% CI: 0.20–0.98; *p* = 0.043) significantly associated with a lower likelihood of undergoing invasive treatment (Table 7). In the STEMI subgroup, none of the variables reached statistical significance in the multivariate model (Table 8).

#### 3.2.2. Discharge Medications

Across the entire ACS cohort, discharge medications significantly differed between treatment strategies. Patients treated conservatively were less likely to receive antiplatelet therapy, RAAS inhibitors, and statins, and more likely to be prescribed oral anticoagulants. There was no difference in beta-blocker prescription (Table 9). Similar trends were seen in the STEMI subgroup, except that angiotensin-converting enzyme (ACE) inhibitors or angiotensin receptor blockers (ARBs) were not significantly different between groups (Table 10). In the NSTE-ACS subgroup, those treated invasively were more frequently discharged on single or dual antiplatelet therapy and ACE inhibitors or ARBs (Table 11).

### 3.3. Outcomes and Survival

#### 3.3.1. Primary and Secondary Outcomes

During the six-month follow-up period, 174 patients (26%) died. The incidence of the primary outcome was significantly higher in the conservatively managed group (37.8% vs. 19.3%, *p* < 0.001; Table 12). After PSM, the six-month mortality remained significantly higher in the conservatively treated patient group (37.2% vs. 26.1%, *p* = 0.013; Table 13). This trend was consistent across both STEMI (49.4% vs. 26.8%, *p* < 0.001; Table 14) and NSTE-ACS (32.1% vs. 10.9%, *p* < 0.001; Table 15) subgroups. No significant differences were observed in secondary outcomes, including recurrent myocardial infarction and stroke, in any group.

#### 3.3.2. Survival Analysis

**Figure 2:**
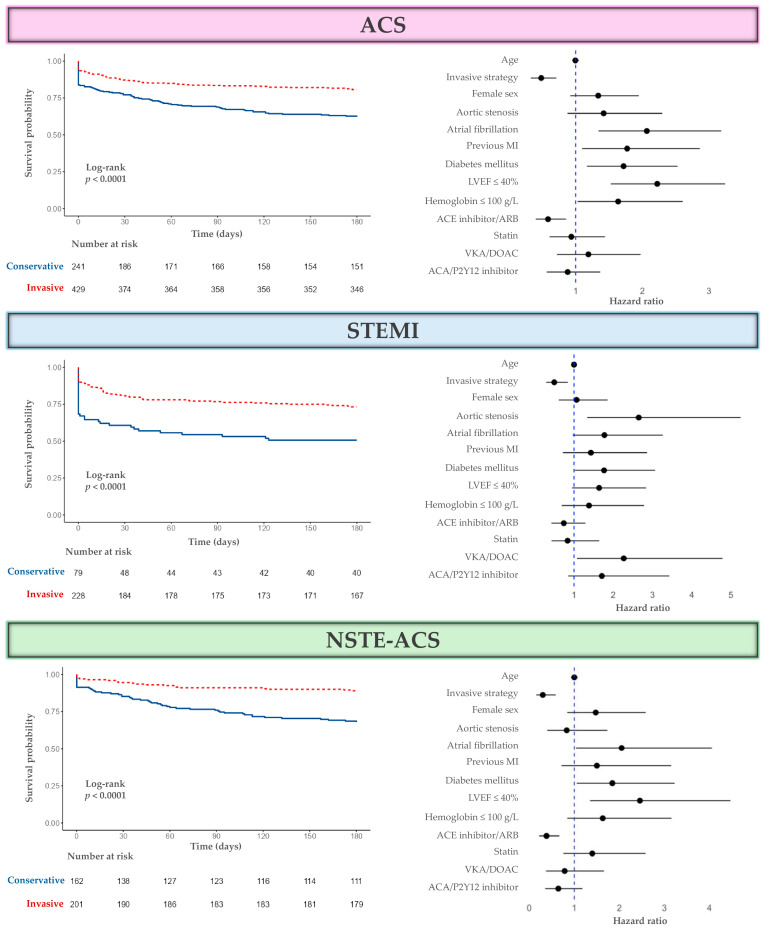
Kaplan–Meier survival curves comparing invasive and conservative treatment strategies and Cox proportional hazards model for six-month survival in patients with ACS (concordance = 0.736 [SE = 0.022]), STEMI (concordance = 0.758 [SE = 0.03]), and NSTE-ACS (concordance = 0.774 [SE = 0.031]).

Multivariable survival analysis showed that invasive treatment was significantly associated with improved six-month survival (Figure 2) across all ACS patients (HR = 0.48; 95% CI: 0.33–0.71; *p* < 0.001), including both STEMI (HR = 0.50; 95% CI: 0.29–0.84; *p* = 0.010) and NSTE-ACS (HR = 0.30; 95% CI: 0.15–0.59; *p* < 0.001). Discharge treatment with renin–angiotensin–aldosterone system inhibitors was also independently associated with improved survival in the NSTE-ACS subgroup, whereas no significant association was observed in the STEMI subgroup.

To formally assess whether the effect of revascularization differed by sex, we included an interaction term between sex (male vs. female) and revascularization in the Cox proportional hazards model. The interaction term was not statistically significant (HR = 0.83; 95% CI: 0.38–1.77; *p* = 0.62), indicating no evidence that the association between revascularization and survival varied between males and females. Therefore, although subgroup analyses suggested differences in treatment allocation by sex, the formal interaction test did not confirm a statistically significant sex-based difference in treatment effect.

In the post-matching data, the invasive treatment strategy (HR = 0.53; 95% CI: 0.35–0.81; *p* = 0.003) and ACE inhibitor or ARB therapy (HR = 0.58; 95% CI: 0.38–1.89; *p* = 0.013) remained significant independent predictors of improved survival, whereas diabetes mellitus (HR = 1.88; 95% CI: 1.23–2.87; *p* = 0.003) and LVEF ≤ 40% (HR = 2.13; 95% CI: 1.40–3.23; *p* < 0.001) were negative predictors (Table 16).

## 4. Discussion

Acute coronary syndrome management in older patients presents unique challenges and considerations due to the physiological changes associated with aging, the presence of multiple comorbidities, and the increased risk of adverse events, including bleeding. Current guidelines rely on foundational studies, such as the FIR trials, but these may not adequately represent the diverse, aging, and highly comorbid patient population seen in today’s clinical settings [33]. Although the ESC guidelines assign a Class I recommendation for invasive management in this population, the level of evidence supporting these recommendations is B, indicating a need for further high-quality studies to confirm the benefits of invasive strategies in elderly patients with ACS [16].

In our real-world observational study, we explored the impact of invasive versus conservative treatment strategies on clinical outcomes in elderly patients with ACS. The findings provide valuable insights into the management of an increasingly prevalent and high-risk patient group. Our results indicate that invasive management was significantly associated with improved six-month survival across the entire ACS population, including both STEMI and NSTE-ACS patients. This aligns with previous observational studies suggesting a survival benefit with invasive strategies in elderly populations, despite the absence of significant mortality reduction in RCTs [22,24,25,26,27,28,29,30].

ACE inhibitors or ARBs therapy at admission, together with the invasive strategy, was significantly associated with improved six-month survival. These findings are consistent with earlier studies demonstrating the impact of ACE inhibitor therapy on reducing mortality and cardiovascular morbidity following myocardial infarction. Outcomes were particularly favorable among patients with left ventricular dysfunction and heart failure. This may help explain why such therapy was a significant predictor of survival in older patients, in whom these high-risk features are more commonly present [34].

The study identified age and elevated CRP levels as independent predictors of conservative management, highlighting the role of frailty and inflammatory status in treatment decision-making. These factors, along with comorbid conditions such as COPD and aortic stenosis, may influence clinicians’ reluctance to pursue invasive interventions in older patients. This cautious approach is understandable given the increased procedural risks in this demographic, as noted in other studies [6,13,14].

Interestingly, our data demonstrate no significant difference in secondary outcomes, such as recurrent myocardial infarction and cerebrovascular insult, between the treatment groups. This finding contrasts with some previous reports of reduced recurrent MI rates with invasive management [22]. However, the lack of difference might be attributed to the study’s limited follow-up duration or the sample size, necessitating further investigations with larger cohorts and extended follow-up periods.

The gender disparity observed in treatment strategies, particularly the lower likelihood of invasive management in women with NSTE-ACS, warrants attention. This reflects broader trends in cardiovascular care where gender biases may affect treatment access and outcomes [9]. Addressing such disparities is crucial for ensuring equitable healthcare delivery. Montoy et al. showed that among NSTEMI patients, 56.3% of men underwent timely angiography compared to only 45.9% of women [35].

Lee et al. showed that women with NSTEMI have higher unadjusted in-hospital and 30 day mortality (e.g., 9.93% vs. 7.10% for NSTEMI). Firstly, the higher unadjusted mortality rates for women suggest potential systemic biases in healthcare delivery. This disparity implies that women might face delays in diagnosis or receive less aggressive treatment compared to men. Upon adjusting for variables such as age and clinical presentation, the mortality differences diminish, which indicates that these disparities are not solely due to biological factors. This adjustment underscores the role of treatment biases, suggesting that healthcare providers may not be fully attuned to the unique presentation of heart conditions in women, leading to suboptimal care. However, it is concerning that even after adjustments, meta-analyses indicate women are at a 2.26 times greater risk for 30 day mortality. This finding suggests that the adjustments may not fully account for all the nuances of gender disparities in treatment and outcomes. It points to the possibility of deeper, systemic issues such as implicit biases in clinical decision-making or differences in the availability and accessibility of care [36].

The underrepresentation of women in clinical trials presents a critical challenge in medical research, leading to treatment protocols that may not fully address the unique health needs of women. Historically, the exclusion of women stemmed from concerns over hormonal variability and potential risks to reproductive health, which inadvertently resulted in a significant data gap. This gap has meant that many treatment guidelines are predominantly informed by male physiology, potentially compromising the safety and effectiveness of treatments for women. Despite recent advancements in increasing women’s participation in clinical trials, these historical biases continue to affect current medical practices. The lack of gender-specific data means that healthcare providers might apply treatment protocols that do not optimally cater to women’s physiological differences. This underlines the importance of continued advocacy for gender-specific research and trials.

As with women, the underrepresentation of elderly populations in clinical trials for ACS presents a significant challenge to optimizing treatment protocols for this age group. Given the higher prevalence of ACS among the elderly, their exclusion from research creates a critical gap in our understanding of how treatments should be tailored to meet their specific needs. One of the primary challenges is the presence of comorbidities and polypharmacy, which complicate trial designs and outcomes. Researchers may exclude elderly participants to maintain trial simplicity, but this leads to data that may not apply to the wider population who suffer from ACS. As a result, current treatment guidelines may inadequately address the unique physiological changes and health conditions common in older adults. Recent efforts to include elderly participants in clinical trials have shown promise, with some studies beginning to specifically target this demographic or modify protocols to accommodate their needs. This shift is crucial, as it acknowledges the importance of generating age-specific data to improve treatment outcomes.

The study by Spadafora et al. presents valuable real-world data comparing one-year outcomes between patients with STEMI and NSTEMI, based on the extensive PRAISE registry. The research addresses an important clinical question and offers useful insights into contemporary ACS management. However, several methodological and interpretative aspects warrant closer scrutiny. The analysis spans a long-time frame (2003–2019), during which significant changes in guideline-based treatment and revascularization strategies occurred. Yet, no stratified analyses or sensitivity checks were performed to assess the potential impact of treatment-era variability on outcomes. This temporal heterogeneity could have influenced both management decisions and prognosis, particularly for NSTEMI patients, who have historically been less aggressively treated. The study also highlights gender-based and comorbidity-based differences in treatment allocation but does not conduct formal statistical interaction testing to confirm whether treatment effects significantly differ across subgroups. Without these analyses, subgroup observations remain speculative and should be interpreted with caution. While the authors conclude that adjusted one-year outcomes between STEMI and NSTEMI patients are similar, the authors do not discuss known issues such as undertreatment of NSTEMI patients, which remains a persistent and clinically relevant problem. This study contributes important observational data from a large international cohort and supports the notion that initial ECG presentations may not fully determine long-term prognosis in ACS patients. Nonetheless, the findings would be more convincing if accompanied by comprehensive reporting of statistical methods, balance diagnostics, model validation, and subgroup interaction testing. Addressing these aspects would significantly enhance the transparency, interpretability, and clinical impact of the study [37].

### Limitations

A key limitation of this study is its retrospective design, which inherently precludes the use of randomization. Treatment allocation was not standardized but instead depended on the clinical judgment of individual physicians, as well as the preferences of patients and their families. To account for this potential selection bias, we performed multivariable regression analyses to explore predictors of invasive treatment and their association with survival outcomes.

Another limitation of this study pertains to the availability of long-term follow-up data. The study involved patient data from two centers; one center provided structured and comprehensive long-term follow-up information, whereas technical constraints at the second center limited systematic collection of data beyond six months. To ensure consistency and comparability of outcomes across the entire cohort, a six-month follow-up period was adopted for all patients. This approach minimized bias related to missing long-term data, although it restricted the assessment of outcomes beyond this time point.

Incomplete clinical documentation also posed a challenge. In a subset of patients, data on laboratory parameters and medication use at admission and discharge were missing. The registry did not systematically capture door-to-balloon time, which limits our ability to analyze this variable. Additionally, several critical factors known to influence decision-making and outcomes in older adults—such as physical frailty, cognitive or sensory impairments, and functional dependence—could not be evaluated, as these are not routinely recorded in medical records.

Comparisons with previous studies were challenging due to varying definitions of invasive management. In many reports, an invasive strategy is defined as coronary angiography with or without revascularization. However, a significant proportion of such patients undergo diagnostic angiography alone, without subsequent percutaneous or surgical intervention. In the context of our study, we classified these cases as conservatively treated, given the absence of therapeutic reperfusion. We believe this approach more accurately reflects the true impact of treatment strategies on clinical outcomes.

We chose all-cause mortality as the primary endpoint, as it better captures the complexity of outcomes in elderly populations. Although cardiovascular mortality may directly reflect the effectiveness of invasive versus conservative strategies in ACS, older patients often present with multiple comorbidities and reduced physiological reserve. These factors substantially influence both treatment selection and survival. If a treatment strategy were to reduce only cardiovascular mortality without improving overall survival, its benefit in this patient population would be of limited clinical relevance.

Future research should focus on identifying subpopulations within the elderly cohort that may derive the most benefit from invasive interventions, potentially incorporating frailty indices or biomarkers of biological aging. Additionally, studies exploring the integration of comprehensive geriatric assessments into ACS management protocols could provide a more holistic understanding of treatment impacts beyond traditional cardiovascular outcomes.

## 5. Conclusions

The invasive strategy was a significant independent positive predictor of six-month survival in all ACS patients, including both STEMI and NSTE-ACS.

Overall, our study underscores the complexity of managing ACS in older adults and highlights the need for personalized treatment strategies that consider the unique physiological and clinical characteristics of this population. Further high-quality RCTs are essential to refine guidelines and optimize care for elderly patients with ACS.

## Figures and Tables

**Table 1:** Demographic, clinical, and laboratory characteristics of ACS patients by treatment strategy.

Parameter	ACS			*p*
	All (*n* = 670)	Invasive Strategy (*n* = 429)	Conservative Strategy (*n* = 241)	
	No. (%) Mean ± SD Median (IQR)	No. (%) Mean ± SD Median (IQR)	No. (%) Mean ± SD Median (IQR)	
Age	83 (81–86)	83 (81–86)	84 (82–87)	<0.001 *
Sex				0.035 *
Woman	339 (50.6%)	204 (47.6%)	135 (56.0%)	
Men	331 (49.4%)	225 (52.4%)	106 (44.0%)	
ACS type				<0.001 *
STEMI	307 (45.8%)	228 (53.1%)	79 (32.8%)	
NSTE-ACS	363 (54.2%)	201 (46.9%)	162 (67.2%)	
BMI (kg/m^2^)	26.5 (23.9–29.4)	26.5 (23.9–29.4)	26.4 (23.6–29.6)	0.594
SBP (mmHg)	135 (120–150)	135 (120–150)	137 ± 25	0.391
DBP (mmHg)	80 (70–88)	78 (70–86)	80 (70–90)	0.118
Pulse (beats/min)	80 (67–90)	78 (66–88)	80 (70–95)	0.003 *
Smoking history	100 (14.9%)	69 (16.1%)	31 (12.9)	0.262
Previous MI	117 (17.5%)	70 (16.3%)	47 (19.5%)	0.297
Previous revascularization	135 (20.1%)	79 (18.4%)	56 (23.2%)	0.135
Previous CVI	77 (11.5%)	45 (10.5%)	32 (13.3%)	0.277
Arterial hypertension	586 (87.5%)	380 (88.6%)	206 (85.5%)	0.245
Diabetes mellitus	214 (31.9%)	145 (33.8%)	69 (28.6%)	0.168
Hyperlipidemia	311 (46.4%)	214 (49.9%)	97 (40.2%)	0.016 *
COPD	27 (4.0%)	15 (3.5%)	12 (5.0%)	0.349
Malignant disease	129 (19.3%)	78 (18.2%)	51 (21.2%)	0.348
Atrial fibrillation	189 (28.2%)	107 (24.9%)	82 (34.0%)	0.012 *
Aortic stenosis	77 (11.5%)	33 (7.7%)	44 (18.3%)	<0.001 *
Mitral regurgitation	85 (12.7%)	52 (12.1%)	33 (13.7%)	0.557
Peripheral artery disease	44 (6.6%)	34 (7.9%)	10 (4.1%)	0.058
Chronic medications ^1^				
ACA/P2Y_12_ inhibitor	217 (33.1%)	125 (29.9%)	92 (38.7%)	0.022 *
ACE inhibitor/ARB	412 (62.8%)	266 (63.6%)	146 (61.3%)	0.559
Beta blocker	305 (46.5%)	173 (41.4%)	132 (55.5%)	<0.001 *
Statin	193 (29.4%)	111 (26.6%)	82 (34.5%)	0.033 *
Blood parameters				
Hemoglobin (g/L)	128 (118–140)	131 (119–141)	124 (112–137)	<0.001 *
Platelets (×10^9^/L)	218 (177–266)	213 (180–260)	223 (173–281)	0.297
Total cholesterol (mmol/L)	4.4 (3.6–5.3)	4.6 (3.8–5.4)	4.1 (3.2–5.1)	<0.001 *
LDL cholesterol (mmol/L)	2.7 (2.0–3.4)	2.9 (2.0–3.6)	2.4 (1.7–3.3)	0.001 *
Triglycerides (mmol/L)	1.2 (1.0–1.6)	1.3 (1.0–1.7)	1.2 (1.0–1.5)	0.051
NT-proBNP (pg/mL)	4923 (1967–12,796)	3979 (1631–10,482)	6296 (2853–16,813)	0.004 *
Creatinine (μmol/L)	103 (82–131)	99 (82–127)	109 (80–144)	0.023 *
eGFR (mL/min/1.73 m^2^)	51 (37–66)	53 (40–66)	46 (31–64)	0.004 *
C-reactive protein (mg/L)	11.3 (3.4–51.7)	8.8 (2.8–33.7)	21.1 (4.9–73.8)	<0.001 *
In-hospital				
Major bleeding	16 (2.4%)	12 (2.8%)	4 (1.7%)	0.355
LVEF ≤ 40%	200 (29.9%)	115 (26.8%)	85 (35.3%)	0.022 *
Cardiogenic shock	37 (5.5%)	21 (4.9%)	16 (6.6%)	0.343

^1^ Data on chronic medication were missing for 14 patients; ACA = acetylsalicylic acid; ACE = angiotensin-converting enzyme; ARB = angiotensin receptor blocker; BMI = body mass index; COPD = chronic obstructive pulmonary disease; CVI = cerebrovascular insult; DBP = diastolic blood pressure; eGFR = estimated glomerular filtration rate; LDL = low-density lipoprotein; LVEF = left ventricular ejection fraction; MI = myocardial infarction; NT-proBNP = N-terminal pro–B-type natriuretic peptide; P2Y_12_ = purinergic receptor type Y, subtype 12; SBP = systolic blood pressure; *: *p* < 0.05.

**Table 2:** Demographic, clinical, and laboratory characteristics of ACS patients by treatment strategy, performed after propensity score matching.

Parameter	ACS			*p*
	All (*n* = 436)	Invasive Strategy (*n* = 218)	Conservative Strategy (*n* = 218)	
	No. (%) Median (IQR)	No. (%) Median (IQR)	No. (%) Median (IQR)	
Age	84 (81–87)	83 (81–87)	84 (82–87)	0.147
Sex				0.700
Woman	240 (55.0%)	122 (56.0%)	118 (54.1%)	
Men	196 (45.0%)	96 (44.0%)	100 (45.9%)	
ACS type				<0.001 *
STEMI	200 (45.9%)	129 (59.2%)	71 (32.6%)	
NSTE-ACS	236 (54.1%)	89 (40.8%)	147 (67.4%)	
BMI (kg/m^2^)	26.1 (23.6–29.4)	26 (23.9–29.0)	26.1 (23.4–29.5)	0.805
SBP (mmHg)	135 (120–150)	130 (118–145)	137 (120–150)	0.030 *
DBP (mmHg)	80 (69–87)	77 (65–85)	80 (70–90)	0.009 *
Pulse (beats/min)	80 (68–90)	80 (66–88)	80 (70–95)	0.076
Smoking history	60 (13.8%)	31 (14.2%)	29 (13.3%)	0.781
Previous MI	79 (18.1%)	32 (14.7%)	47 (21.6%)	0.062
Previous revascularization	89 (20.4%)	35 (16.1%)	54 (24.8%)	0.024 *
Previous CVI	55 (12.6%)	29 (13.3%)	26 (11.9%)	0.665
Arterial hypertension	381 (87.4%)	192 (88.1%)	189 (86.7%)	0.665
Diabetes mellitus	143 (32.8%)	77 (35.3%)	66 (30.3%)	0.262
Hyperlipidemia	189 (43.3%)	98 (45.0%)	91 (41.7%)	0.499
COPD	20 (4.6%)	9 (4.1%)	11 (5.0%)	0.647
Malignant disease	87 (20.0%)	42 (19.3%)	45 (20.6%)	0.719
Atrial fibrillation	143 (32.8%)	72 (33.0%)	71 (32.6%)	0.919
Aortic stenosis	65 (14.9%)	31 (14.2%)	34 (15.6%)	0.687
Mitral regurgitation	62 (14.2%)	35 (16.1%)	27 (12.4%)	0.273
Peripheral artery disease	23 (5.3%)	13 (6.0%)	10 (4.6%)	0.520
Chronic medications ^1^				
ACA/P2Y_12_ inhibitor	143 (32.8%)	58 (26.6%)	85 (39.0%)	0.006 *
ACE inhibitor/ARB	263 (60.3%)	130 (59.6%)	133 (61.0%)	0.769
Beta blocker	214 (49.1%)	91 (41.7%)	123 (56.4%)	0.002 *
Statin	125 (28.7%)	49 (22.5%)	76 (34.9%)	0.004 *
Blood parameters				
Hemoglobin (g/L)	127 (115–139)	128 (117–139)	125 (114–138)	0.250
Platelets (×10^9^/L)	220 (175–273)	218 (177–267)	224 (174–281)	0.435
Total cholesterol (mmol/L)	4.3 (3.5–5.2)	4.5 (3.7–5.2)	4.1 (3.2–5.1)	0.012 *
LDL cholesterol (mmol/L)	2.5 (1.9–3.3)	2.7 (2.1–3.4)	2.4 (1.7–3.3)	0.029 *
Triglycerides (mmol/L)	1.3 (1.0–1.6)	1.3 (1.0–1.8)	1.2 (1.0–1.5)	0.065
NT-proBNP (pg/mL)	5299 (2463–14,353)	5031 (2350–14,353)	5463 (2655–15,103)	0.549
Creatinine (μmol/L)	106 (80–137)	99 (81–133)	108 (80–144)	0.259
eGFR (mL/min/1.73 m^2^)	49 (34–66)	51 (35–66)	47 (32–66)	0.368
C-reactive protein (mg/L)	14.3 (4.0–61.7)	11.8 (3.6–48.7)	18.0 (4.6–75.6)	0.029 *
In-hospital				
Major bleeding	8 (1.8%)	4 (1.8%)	4 (1.8%)	1.000
LVEF ≤ 40%	150 (34.4%)	78 (35.8%)	72 (33.0%)	0.545
Cardiogenic shock	29 (6.7%)	14 (6.4%)	15 (6.9%)	0.848

^1^ Data on chronic medication were missing for 14 patients; ACA = acetylsalicylic acid; ACE = angiotensin-converting enzyme; ARB = angiotensin receptor blocker; BMI = body mass index; COPD = chronic obstructive pulmonary disease; CVI = cerebrovascular insult; DBP = diastolic blood pressure; eGFR = estimated glomerular filtration rate; LDL = low-density lipoprotein; LVEF = left ventricular ejection fraction; MI = myocardial infarction; NT-proBNP = N-terminal pro–B-type natriuretic peptide; P2Y_12_ = purinergic receptor type Y, subtype 12; SBP = systolic blood pressure; *: *p* < 0.05.

**Table 3:** Demographic and clinical characteristics, treatment strategies, and outcomes in ACS patients according to ACS type.

Parameter	ACS			*p*
	All (*n* = 670)	STEMI (*n* = 307)	NSTE-ACS (*n* = 363)	
	No. (%) Median (IQR)	No. (%) Median (IQR)	No. (%) Median (IQR)	
Age	83 (81–86)	83 (81–87)	83 (81–86)	0.842
Sex				<0.001 *
Woman	339 (50.6%)	188 (61.2%)	151(41.6%)	
Men	331 (49.4%)	119 (38.8%)	212 (58.4%)	
Smoking history	100 (14.9%)	44 (14.3%)	56 (15.4%)	0.692
Previous MI	117 (17.5%)	34 (11.1%)	83 (22.9%)	<0.001 *
Previous revascularization	135 (20.1%)	33 (10.7%)	102 (28.1%)	<0.001 *
Previous CVI	77 (11.5%)	37 (12.1%)	40 (11.0%)	0.676
Arterial hypertension	586 (87.6%)	265 (86.3%)	321 (88.4%)	0.411
Diabetes mellitus	214 (31.9%)	95 (30.9%)	119 (32.8%)	0.611
Hyperlipidemia	311 (46.4%)	130 (42.3%)	181 (49.9%)	0.052
COPD	27 (4.0%)	10 (3.3%)	17 (4.7%)	0.350
Malignant disease	129 (19.3%)	51 (16.6%)	78 (21.5%)	0.111
Atrial fibrillation	189 (28.2%)	94 (30.6%)	95 (26.2%)	0.202
Aortic stenosis	77 (11.5%)	21 (6.8%)	56 (15.4%)	<0.001 *
Mitral regurgitation	85 (12.7%)	36 (11.7%)	49 (13.5%)	0.492
Peripheral artery disease	44 (6.6%)	17 (5.5%)	27 (7.4%)	0.322
In-hospital				
Major bleeding	16 (2.4%)	8 (2.6%)	8 (2.2%)	0.734
LVEF ≤ 40%	200 (29.9%)	114 (37.1%)	86 (23.7%)	<0.001 *
Cardiogenic shock	37 (5.5%)	34 (11.1%)	3 (0.8%)	<0.001 *
Treatment strategy				<0.001 *
Invasive strategy	429 (64.0%)	228 (74.3%)	201 (55.4%)	
Conservative strategy	241 (36.0%)	79 (25.7%)	162 (44.6%)	
Primary outcomes All-cause mortality				
In-hospital mortality	67 (10.0%)	48 (15.6%)	19 (5.2%)	<0.001 *
Thirty-day mortality	110 (16.4%)	75 (24.4%)	35 (9.6%)	<0.001 *
Six-month mortality	174 (26.0%)	100 (32.6%)	74 (20.4%)	<0.001 *
Secondary outcomes				
Recurrent MI	17 (2.5%)	6 (2.0%)	11 (3.0%)	0.378
CVI	8 (1.2%)	3 (1.0%)	5 (1.4%)	0.635

COPD = chronic obstructive pulmonary disease; CVI = cerebrovascular insult; LVEF = left ventricular ejection fraction; MI = myocardial infarction; *: *p* < 0.05.

**Table 4:** Demographic, clinical, and laboratory characteristics of STEMI patients by treatment strategy.

Parameter	STEMI			*p*
	All (*n* = 307)	Invasive Strategy (*n* = 228)	Conservative Strategy (*n* = 79)	
	No. (%) Mean ± SD Median (IQR)	No. (%) Mean ± SD Median (IQR)	No. (%) Mean ± SD Median (IQR)	
Age	83 (81–87)	83 (81–86)	84 (82–87)	0.038 *
Sex				0.073
Woman	188 (61.2%)	133 (58.3%)	55 (69.6%)	
Men	119 (38.8%)	95 (41.7%)	24 (30.4%)	
BMI (kg/m^2^)	26.7 (23.9–29.4)	26.7 (23.9–29.4)	26.7 (23.8–29.6)	0.768
SBP (mmHg)	130 ± 25	129 ± 25	132 ± 25	0.350
DBP (mmHg)	77 (68–85)	75 (65–85)	80 ± 14	0.019 *
Pulse (beats/min)	80 (68–90)	80 (66–90)	85 ± 20	0.069
Smoking history	44 (14.3%)	36 (15.8%)	8 (10.1%)	0.216
Previous MI	34 (11.1%)	26 (11.4%)	8 (10.1%)	0.755
Previous revascularization	33 (10.7%)	24 (10.5%)	9 (11.4%)	0.830
Previous CVI	37 (12.1%)	26 (11.4%)	11 (13.9%)	0.553
Arterial hypertension	265 (86.3%)	199 (87.3%)	66 (83.5%)	0.405
Diabetes mellitus	95 (30.9%)	72 (31.6%)	23 (29.1%)	0.683
Hyperlipidemia	130 (42.3%)	100 (43.9%)	30 (38.0%)	0.362
COPD	10 (3.3%)	10 (4.4%)	0 (0.0%)	0.058
Malignant disease	51 (16.6%)	39 (17.1%)	12 (15.2)	0.693
Atrial fibrillation	94 (30.6%)	60 (26.3%)	34 (43.0%)	0.005 *
Aortic stenosis	21 (6.8%)	13 (5.7%)	8 (10.1%)	0.179
Mitral regurgitation	36 (11.7%)	26 (11.4%)	10 (12.7%)	0.765
Peripheral artery disease	17 (5.5%)	14 (6.1%)	3 (3.8%)	0.433
Chronic medications ^1^				
ACA/P2Y_12_ inhibitor	69 (23.2%)	47 (21.4%)	22 (28.6%)	0.197
ACE inhibitor/ARB	162 (54.5%)	117 (53.2%)	45 (58.4%)	0.425
Beta blocker	115 (38.7%)	76 (34.5%)	39 (50.6%)	0.013 *
Statin	61 (20.5%)	43 (19.5%)	18 (23.4%)	0.474
Blood parameters				
Hemoglobin (g/L)	128 (119–140)	128 (119–140)	125 (118–137)	0.302
Platelets (×10^9^/L)	229 (187–277)	224 (187–269)	240 (190–294)	0.170
Total cholesterol (mmol/L)	4.7 ± 1.3	4.9 ± 1.3	4.2 ± 1.3	0.003 *
LDL cholesterol (mmol/L)	2.9 (2.2–3.5)	3.0 (2.3–3.8)	2.6 ± 1.1	0.010 *
Triglycerides (mmol/L)	1.2 (1.0–1.6)	(1.0–1.6)	1.1 (1.0–1.4)	0.266
NT-proBNP (pg/mL)	5887 (3225–15,106)	5473 (2853–13,447)	9969 (5113–19,216)	0.006 *
Creatinine (μmol/L)	102 (81–130)	99 (81–127)	110 (82–145)	0.073
eGFR (mL/min/1.73 m^2^)	49 (36–64)	53 (39–66)	44 (30–59)	0.007 *
C-reactive protein (mg/L)	15.5 (5.0–68.1)	11.7 (4.0–48.7)	46.3 (9.2–99.8)	<0.001 *
In-hospital				
Major bleeding	8 (2.6%)	7 (3.1%)	1 (1.3%)	0.386
LVEF ≤ 40%	114 (37.1%)	76 (33.3%)	38 (48.1%)	0.019 *
Cardiogenic shock	34 (11.1%)	20 (8.8%)	14 (17.7%)	0.029 *

^1^ Data on chronic medication were missing for 10 patients; ACA = acetylsalicylic acid; ACE = angiotensin-converting enzyme; ARB = angiotensin receptor blocker; BMI = body mass index; COPD = chronic obstructive pulmonary disease; CVI = cerebrovascular insult; DBP = diastolic blood pressure; eGFR = estimated glomerular filtration rate; LDL = low-density lipoprotein; LVEF = left ventricular ejection fraction; MI = myocardial infarction; NT-proBNP = N-terminal pro–B-type natriuretic peptide; P2Y_12_ = purinergic receptor type Y, subtype 12; SBP = systolic blood pressure; *: *p* < 0.05.

**Table 5:** Demographic, clinical, and laboratory characteristics of NSTE-ACS patients by treatment strategy.

Parameter	NSTE-ACS			*p*
	All (*n* = 363)	Invasive Strategy (*n* = 201)	Conservative Strategy (*n* = 162)	
	No. (%) Mean ± SD Median (IQR)	No. (%) Mean ± SD Median (IQR)	No. (%) Mean ± SD Median (IQR)	
Age	83 (81–86)	83 (81–85)	84 (82–87)	<0.001 *
Sex				0.007 *
Woman	151 (41.6%)	71 (35.3%)	80 (49.4%)	
Men	212 (58.4%)	130 (64.7%)	82 (50.6%)	
BMI (kg/m^2^)	26.4 (23.8–29.6)	26.4 (24.0–29.5)	26.4 ± 4.2	0.419
SBP (mmHg)	140 (125–152)	140 (127–153)	140 (120–151)	0.293
DBP (mmHg)	80 (70–90)	80 (70–90)	79 ± 13	0.598
Pulse (beats/min)	77 (67–90)	75 (65–87)	80 (70–94)	0.004 *
Smoking history	56 (15.4%)	33 (16.4%)	23 (14.2%)	0.560
Previous MI	83 (22.9%)	44 (21.9%)	39 (24.1%)	0.622
Previous revascularization	102 (28.1%)	55 (27.4%)	47 (29.0%)	0.728
Previous CVI	40 (11.0%)	19 (9.5%)	21 (13.0%)	0.288
Arterial hypertension	321 (88.4%)	181 (90.0%)	140 (86.4%)	0.282
Diabetes mellitus	119 (32.8%)	73 (36.3%)	46 (28.4%)	0.110
Hyperlipidemia	181 (49.9%)	114 (56.7%)	67 (41.4%)	0.004 *
COPD	17 (4.7%)	5 (2.5%)	12 (7.4%)	0.027 *
Malignant disease	78 (21.5%)	39 (19.4%)	39 (24.1%)	0.281
Atrial fibrillation	95 (26.2%)	47 (23.4%)	48 (29.6%)	0.178
Aortic stenosis	56 (15.4%)	20 (10.0%)	36 (22.2%)	0.001 *
Mitral regurgitation	49 (13.5%)	26 (12.9%)	23 (14.2%)	0.726
Peripheral artery disease	27 (7.4%)	20 (10.0%)	7 (4.3%)	0.042 *
Chronic medications ^1^				
ACA/P2Y_12_ inhibitor	148 (41.2%)	78 (39.4%)	70 (43.5%)	0.434
ACE inhibitor/ARB	250 (69.9%)	149 (75.3%)	101 (62.7%)	0.010 *
Beta blocker	190 (52.9%)	97 (49.0%)	93 (57.8%)	0.098
Statin	132 (36.8%)	68 (34.3%)	64 (39.8%)	0.291
Blood parameters				
Hemoglobin (g/L)	129 (115–140)	132 (120–141)	122 ± 22	<0.001 *
Platelets (×10^9^/L)	205 (169–257)	202 (167–249)	213 (169–271)	0.206
Total cholesterol (mmol/L)	4.3 (3.4–5.2)	4.4 (3.6–5.2)	4.1 (3.2–5.2)	0.135
LDL cholesterol (mmol/L)	2.5 (1.8–3.4)	2.6 (1.8–3.4)	2.4 (1.7–3.3)	0.185
Triglycerides (mmol/L)	1.3 (1.0–1.7)	1.3 (1.0–1.8)	1.2 (1.0–1.6)	0.051
NT-proBNP (pg/mL)	3407 (1246–11,120)	2350 (799–5806)	5029 (1794–13,393)	0.002 *
Creatinine (μmol/L)	105 (82–133)	97 (84–126)	108 (79–144)	0.190
eGFR (mL/min/1.73 m^2^)	52 (37–67)	54 ± 19	50 (32–67)	0.093
C-reactive protein (mg/L)	8.8 (2.4–35.9)	5.2 (1.9–22.6)	15.1 (3.9–58.4)	<0.001 *
In-hospital				
Major bleeding	8 (2.2%)	5 (2.5%)	3 (1.9%)	0.682
LVEF ≤ 40%	86 (23.7%)	39 (19.4%)	47 (29.0%)	0.032 *
Cardiogenic shock	3 (0.8%)	1 (0.5%)	2 (1.2%)	0.441

^1^ Data on chronic medication were missing for 4 patients; ACA = acetylsalicylic acid; ACE = angiotensin-converting enzyme; ARB = angiotensin receptor blocker; BMI = body mass index; COPD = chronic obstructive pulmonary disease; CVI = cerebrovascular insult; DBP = diastolic blood pressure; eGFR = estimated glomerular filtration rate; LDL = low-density lipoprotein; LVEF = left ventricular ejection fraction; MI = myocardial infarction; NT-proBNP = N-terminal pro–B-type natriuretic peptide; P2Y_12_ = purinergic receptor type Y, subtype 12; SBP = systolic blood pressure; *: *p* < 0.05.

**Table 6:** Results of multivariate logistic regression analysis assessing independent predictors of invasive treatment selection in ACS patients.

Variable	OR	95% CI	*p*
Age	1.10	1.03–1.18	0.005 *
Pulse ^1^	1.00	0.99–1.02	0.641
NT-proBNP ^1^	1.00	1.00–1.00	0.980
Creatinine ^1^	1.00	1.00–1.00	0.770
C-reactive protein ^1^	1.01	1.00–1.01	0.012 *
Female sex	0.74	0.44–1.23	0.242
Hyperlipidemia	1.38	0.80–2.39	0.245
ACA/P2Y_12_ inhibitor ^2^	0.69	0.40–1.19	0.183
Beta blocker ^2^	0.64	0.38–1.06	0.084
Statin ^2^	0.86	0.48–1.57	0.632
Atrial fibrillation	0.83	0.49–1.40	0.475
Aortic stenosis	0.59	0.28–1.22	0.153
Peripheral artery disease	4.35	0.73–25.76	0.106
Hemoglobin ≤ 100 g/L ^1^	0.70	0.31–1.57	0.391
LVEF ≤ 40% ^3^	0.69	0.40–1.19	0.186

Area under the curve = 0.707; R^2^_N_ = 0.154; overall model test (χ^2^ = 39.4, df = 15, *p* < 0.001); ^1^ values obtained during initial clinical assessment and laboratory evaluation; ^2^ admission medication; ^3^ parameter measured during hospitalization; ACA = acetylsalicylic acid; LVEF = left ventricular ejection fraction; NT-proBNP = N-terminal pro–B-type natriuretic peptide; P2Y_12_ = purinergic receptor type Y, subtype 12; *: *p* < 0.05.

**Table 7:** Results of multivariate logistic regression analysis assessing independent predictors of invasive treatment selection in NSTE-ACS patients.

Variable	OR	95% CI	*p*
Age	1.11	1.03–1.19	0.004 *
Pulse ^1^	1.01	1.00–1.03	0.085
C-reactive protein ^1^	1.00	1.00–1.01	0.087
Female sex	0.64	0.39–1.04	0.072
Hyperlipidemia	1.29	0.78–2.14	0.320
ACE inhibitor/ARB ^2^	1.32	0.77–2.27	0.317
Beta blocker ^2^	0.68	0.41–1.11	0.121
Aortic stenosis	0.45	0.23–0.87	0.018 *
COPD	0.29	0.09–0.92	0.035 *
Peripheral artery disease	1.68	0.58–4.89	0.340
Hemoglobin ≤ 100 g/L ^1^	0.44	0.20–0.98	0.043 *
LVEF ≤ 40% ^3^	0.62	0.35–1.10	0.102

Area under the curve = 0.729; R^2^_N_ = 0.219; overall model test (χ^2^ = 59.8, df = 12, *p* < 0.001); ^1^ values obtained during initial clinical assessment and laboratory evaluation; ^2^ admission medication; ^3^ parameter measured during hospitalization; ACE = angiotensin-converting enzyme; ARB = angiotensin receptor blocker; COPD = chronic obstructive pulmonary disease; LVEF = left ventricular ejection fraction; *: *p* < 0.05.

**Table 8:** Results of multivariate logistic regression analysis assessing independent predictors of invasive treatment selection in STEMI patients.

Variable	OR	95% CI	*p*
Age	1.13	1.00–1.28	0.057
DBP ^1^	1.03	1.00–1.07	0.135
Pulse ^1^	1.00	0.97–1.02	0.740
LDL cholesterol ^1^	0.76	0.47–1.22	0.253
NT-proBNP ^1^	1.00	1.00–1.00	0.320
Creatinine ^1^	1.00	0.99–1.01	0.449
C-reactive protein ^1^	1.01	1.00–1.02	0.096
Female sex	0.66	0.23–1.91	0.441
Beta blocker ^2^	1.04	0.39–2.81	0.934
Atrial fibrillation	0.58	0.20–1.65	0.309
LVEF ≤ 40% ^3^	1.30	0.41–4.10	0.665

Area under the curve = 0.777; R^2^_N_ = 0.244; overall model test (χ^2^ = 20.3, df = 11, *p* = 0.041); ^1^ values obtained during initial clinical assessment and laboratory evaluation; ^2^ admission medication; ^3^ parameter measured during hospitalization; DBP = diastolic blood pressure; LDL = low-density lipoprotein; LVEF = left ventricular ejection fraction; NT-proBNP = N-terminal pro–B-type natriuretic peptide.

**Table 9:** Discharge medication profiles of ACS patients according to treatment strategy.

Parameter	ACS			*p*
	All ^1^ (*n* = 603)	Invasive Strategy (*n* = 401)	Conservative Strategy (*n* = 202)	
	No. (%)	No. (%)	No. (%)	
Discharge medication				
ACA/P2Y_12_ inhibitor	559 (92.7%)	395 (98.5%)	164 (81.2%)	<0.001 *
ACE inhibitor/ARB	485 (80.4%)	334 (83.3%)	151 (74.8%)	0.013 *
Beta blocker	497 (82.4%)	329 (82.0%)	168 (83.2%)	0.732
Statin	574 (95.2%)	391 (97.5%)	183 (90.6%)	<0.001 *
VKA/DOAC	151 (25.0%)	83 (20.7%)	68 (33.7%)	<0.001 *

^1^ A total of 67 patients died during hospitalization; ACA = acetylsalicylic acid; ACE = angiotensin-converting enzyme; ARB = angiotensin receptor blocker; DOAC = direct oral anticoagulant; P2Y_12_ = purinergic receptor type Y, subtype 12; VKA = vitamin K antagonist; *: *p* < 0.05.

**Table 10:** Discharge medication profiles of STEMI patients according to treatment strategy.

Parameter	STEMI			*p*
	All ^1^ (*n* = 259)	Invasive Strategy (*n* = 205)	Conservative Strategy (*n* = 54)	
	No. (%)	No. (%)	No. (%)	
Discharge medication				
ACA/P2Y_12_ inhibitor	244 (94.2%)	202 (98.5%)	42 (77.8%)	<0.001 *
ACE inhibitor/ARB	203 (78.4%)	165 (80.5%)	38 (70.4%)	0.108
Beta blocker	207 (79.9%)	164 (80.0%)	43 (79.6%)	0.952
Statin	255 (98.5%)	205 (100.0%)	50 (92.6%)	<0.001 *
VKA/DOAC	70 (27.0%)	44 (21.5%)	26 (48.1%)	<0.001 *

^1^ A total of 48 patients died during hospitalization; ACA = acetylsalicylic acid; ACE = angiotensin-converting enzyme; ARB = angiotensin receptor blocker; DOAC = direct oral anticoagulant; P2Y_12_ = purinergic receptor type Y, subtype 12; VKA = vitamin K antagonist; *: *p* < 0.05.

**Table 11:** Discharge medication profiles of NSTE-ACS patients according to treatment strategy.

Parameter	NSTE-ACS			*p*
	All ^1^ (*n* = 344)	Invasive Strategy (*n* = 196)	Conservative Strategy (*n* = 148)	
	No. (%)	No. (%)	No. (%)	
Discharge medication				
ACA/P2Y_12_ inhibitor	315 (91.6%)	193 (98.5%)	122 (82.4%)	<0.001 *
ACE inhibitor/ARB	282 (82.0%)	169 (86.2%)	113 (76.4%)	0.018 *
Beta blocker	290 (84.3%)	165 (84.2%)	125 (84.5%)	0.944
Statin	319 (92.7%)	186 (94.9%)	133 (89.9%)	0.075
VKA/DOAC	81 (23.5%)	39 (19.9%)	42 (28.4%)	0.066

^1^ A total of 19 patients died during hospitalization; ACA = acetylsalicylic acid; ACE = angiotensin-converting enzyme; ARB = angiotensin receptor blocker; DOAC = direct oral anticoagulant; P2Y_12_ = purinergic receptor type Y, subtype 12; VKA = vitamin K antagonist; *: *p* < 0.05.

**Table 12:** Primary and secondary outcomes in ACS patients stratified by treatment strategy.

Parameter	ACS			*p*
	All (*n* = 670)	Invasive Strategy (*n* = 429)	Conservative Strategy (*n* = 241)	
	No. (%)	No. (%)	No. (%)	
Primary outcomes All-cause mortality				
In-hospital mortality	67 (10.0%)	28 (6.5%)	39 (16.2%)	<0.001 *
Thirty-day mortality	110 (16.4%)	55 (12.8%)	55 (22.8%)	<0.001 *
Six-month mortality	174 (26.0%)	83 (19.3%)	91 (37.8%)	<0.001 *
Secondary outcomes				
Recurrent MI	17 (2.5%)	12 (2.8%)	5 (2.1%)	0.586
CVI	8 (1.2%)	5 (1.2%)	3 (1.2%)	0.928
MACE	184 (27.5%)	89 (20.7%)	95 (39.4%)	<0.001 *

CVI = cerebrovascular insult; MI = myocardial infarction; MACE = major adverse cardiovascular event; *: *p* < 0.05.

**Table 13:** Primary and secondary outcomes in ACS patients stratified by treatment strategy, performed after propensity score matching.

Parameter	ACS			*p*
	All (*n* = 436)	Invasive Strategy (*n* = 218)	Conservative Strategy (*n* = 218)	
	No. (%)	No. (%)	No. (%)	
Primary outcomes All-cause mortality				
In-hospital mortality	56 (12.8%)	21 (9.6%)	35 (16.1%)	0.045 *
Thirty-day mortality	88 (20.2%)	39 (17.9%)	49 (22.9%)	0.233
Six-month mortality	138 (31.7%)	57 (26.1%)	81 (37.2%)	0.013 *
Secondary outcomes				
Recurrent MI	14 (3.2%)	9 (4.1%)	5 (2.3%)	0.277
CVI	4 (0.9%)	1 (0.5%)	3 (1.4%)	0.315
MACE	145 (33.3%)	60 (27.5%)	85 (39.0%)	0.011 *

CVI = cerebrovascular insult; MI = myocardial infarction; MACE = major adverse cardiovascular event; *: *p* < 0.05.

**Table 14:** Primary and secondary outcomes in STEMI patients stratified by treatment strategy.

Parameter	STEMI			*p*
	All (*n* = 307)	Invasive Strategy (*n* = 228)	Conservative Strategy (*n* = 79)	
	No. (%)	No. (%)	No. (%)	
Primary outcomes All-cause mortality				
In-hospital mortality	48 (15.6%)	23 (10.1%)	25 (31.6%)	<0.001 *
Thirty-day mortality	75 (24.4%)	44 (19.3%)	31 (39.2%)	<0.001 *
Six-month mortality	100 (32.6%)	61 (26.8%)	39 (49.4%)	<0.001 *
Secondary outcomes				
Recurrent MI	6 (2.0%)	4 (1.8%)	2 (2.5%)	0.667
CVI	3 (1.0%)	3 (1.3%)	0 (0.0%)	0.306
MACE	102 (33.2%)	63 (27.6%)	39 (49.4%)	<0.001 *

CVI = cerebrovascular insult; MI = myocardial infarction; MACE = major adverse cardiovascular event; *: *p* < 0.05.

**Table 15:** Primary and secondary outcomes in NSTE-ACS patients stratified by treatment strategy.

Parameter	NSTE-ACS			*p*
	All (*n* = 363)	Invasive Strategy (*n* = 201)	Conservative Strategy (*n* = 162)	
	No. (%)	No. (%)	No. (%)	
Primary outcomes All-cause mortality				
In-hospital mortality	19 (2.5%)	5 (2.5%)	14 (8.6%)	0.009 *
Thirty-day mortality	35 (9.6%)	11 (5.5%)	24 (14.8%)	0.003 *
Six-month mortality	74 (20.4%)	22 (10.9%)	52 (32.1%)	<0.001 *
Secondary outcomes				
Recurrent MI	11 (3.0%)	8 (4.0%)	3 (1.9%)	0.240
CVI	5 (1.4%)	2 (1.0%)	3 (1.9%)	0.486
MACE	82 (22.6%)	26 (12.9%)	56 (34.6%)	<0.001 *

CVI = cerebrovascular insult; MI = myocardial infarction; MACE = major adverse cardiovascular event; *: *p* < 0.05.

**Table 16:** Cox proportional hazards model for six-month survival in patients with ACS, performed after propensity score matching.

Parameter	ACS					
		Univariate		Multivariate		
	HR	95% CI	*p*	HR	95% CI	*p*
Age	1.02	0.97–1.07	0.442	1.00	0.95–1.06	0.961
Invasive strategy	0.59	0.39–0.90	0.013 *	0.53	0.35–0.81	0.003 *
Female sex	1.15	0.77–1.72	0.483	1.30	0.86–1.97	0.217
Aortic stenosis	1.30	0.78–2.17	0.307	1.45	0.85–2.48	0.173
Atrial fibrillation	1.33	0.89–1.99	0.162	1.59	0.99–2.55	0.054
Previous MI	0.84	0.50–1.39	0.495	0.82	0.46–1.46	0.492
Diabetes mellitus	1.83	1.22–2.72	0.003 *	1.88	1.23–2.87	0.003 *
LVEF ≤ 40%	1.92	1.29–2.85	0.001 *	2.13	1.40–3.23	<0.001 *
Hemoglobin ≤ 100 g/L	1.84	1.13–3.01	0.015 *	1.46	0.87–2.46	0.154
ACE inhibitor/ARB ^1^	0.61	0.41–0.91	0.015 *	0.58	0.38–0.89	0.013 *
Statin ^1^	0.87	0.56–1.33	0.512	0.92	0.55–1.53	0.742
VKA/DOAC ^1^	1.41	0.88–2.27	0.155	1.10	0.63–1.93	0.738
ACA/P2Y_12_ inhibitor ^1^	0.88	0.58–1.35	0.570	0.97	0.59–1.58	0.892

^1^ Admission medication; concordance = 0.685 [SE = 0.027]; ACA = acetylsalicylic acid; ACE = angiotensin-converting enzyme; ARB = angiotensin receptor blocker; DOAC = direct oral anticoagulant; LVEF = left ventricular ejection fraction; P2Y_12_ = purinergic receptor type Y, subtype 12; VKA = vitamin K antagonist; *: *p* < 0.05.

## Data Availability

The data presented in this study is available on request from the corresponding author.

## Abbreviations

The following abbreviations are used in this manuscript:

ACE	Angiotensin-converting enzyme
ACS	Acute coronary syndrome
ARB	Angiotensin receptor blocker
CI	Confidence interval
COPD	Chronic obstructive pulmonary disease
CRP	C-reactive protein
CVI	Cerebrovascular insult
ESC	European Society of Cardiology
HR	Hazard ratio
IQR	Interquartile range
LDL	Low-density lipoprotein
LVEF	Left ventricular ejection fraction
MACE	Major adverse cardiovascular event
MI	Myocardial infarction
NSTE-ACS	Non-ST-elevation acute coronary syndrome
NSTEMI	Non-ST-elevation myocardial infarction
NT-proBNP	N-terminal pro–B-type natriuretic peptide
OR	Odds ratio
PSM	Propensity score matching
RCT	Randomized controlled trial
SD	Standard deviation
STEMI	ST-elevation myocardial infarction
